# Health Status and Health Care Needs of Drought-Related Migrants in the Horn of Africa—A Qualitative Investigation

**DOI:** 10.3390/ijerph17165917

**Published:** 2020-08-14

**Authors:** Kristina Lindvall, John Kinsman, Atakelti Abraha, Abdirisak Dalmar, Mohamed Farah Abdullahi, Hagos Godefay, Lelekoitien Lerenten Thomas, Mohamed Osman Mohamoud, Bile Khalif Mohamud, Jairus Musumba, Barbara Schumann

**Affiliations:** 1 Department of Epidemiology and Global Health, Faculty of Medicine, Umeå University, 901 87 Umeå, Sweden; john.kinsman@umu.se (J.K.); barbara.schumann@umu.se (B.S.); 2 Department of Public Health Sciences, Global Health (Division of International Health—IHCAR), Karolinska Institutet, 171 77 Stockholm, Sweden; 3 Health Insurance Agency, Federal Ministry of Health, 1000 Addis Ababa, Ethiopia; atakelti@yahoo.com; 4 Somali Disaster Resilience Institute (SDRI), Mogadishu, Somalia; drdalmar@yahoo.co.uk; 5 Department of Research and Development, Puntland University of Science and Technology, Galkayo, Puntland, Somalia; mfchilm026@googlemail.com; 6 Tigray Regional Health Bureau, Tigray, 07 Mekelle, Ethiopia; hgodefay@yahoo.com; 7 Climate Change Directorate, Ministry of Environment and Forestry, 00100 Nairobi, Kenya; lerenten12@gmail.com; 8 Sadar Institute, Borama, Somalia; othmansom@gmail.com; 9 Support to Health Policy and System Development with Agenda for Research, Federal Ministry of Health, Mogadishu, Somalia; khalif.bilemohamud@gmail.com; 10 Somali Swedish Researchers’ Association, 162 46 Stockholm, Sweden; 11 Department of Public Health, Nairobi City County Government, 00400 Nairobi, Kenya; jairus_musumba@yahoo.com

**Keywords:** climate change, drought, migration, displacement, health, health care, Horn of Africa, Somalia, Ethiopia, Kenya, knowledge needs

## Abstract

Somalia, Kenya and Ethiopia, situated in the Horn of Africa, are highly vulnerable to climate change, which manifests itself through increasing temperatures, erratic rains and prolonged droughts. Millions of people have to flee from droughts or floods either as cross-border refugees or as internally displaced persons (IDPs). The aim of this study was to identify knowledge status and gaps regarding public health consequences of large-scale displacement in these countries. After a scoping review, we conducted qualitative in-depth interviews during 2018 with 39 stakeholders from different disciplines and agencies in these three countries. A validation workshop was held with a selection of 13 interviewees and four project partners. Malnutrition and a lack of vaccination of displaced people are well-known challenges, while mental health problems and gender-based violence (GBV) are less visible to stakeholders. In particular, the needs of IDPs are not well understood. The treatment of mental health and GBV is insufficient, and IDPs have inadequate access to essential health services in refugee camps. Needs assessment and program evaluations with a patients’ perspective are either lacking or inadequate in most situations. The Horn of Africa is facing chronic food insecurity, poor population health and mass displacement. IDPs are an underserved group, and mental health services are lacking. A development approach is necessary that moves beyond emergency responses to the building of long-term resilience, the provision of livelihood support and protection to reduce displacement by droughts.

## 1. Introduction

In many regions across the globe, climate change is leading to an increase in the severity and frequency of extreme weather, including rapid onset events (e.g., heat waves and heavy rainfall) and slow onset events such as droughts. Both rapid and slow onset climate-related hazards are projected to become more common in the future [1,2]. In East Africa, temperatures have increased over the last 50 years and more extreme heat events have been observed [3]. Average rainfall during the long rain season (March to June) and during the boreal summer (June to September) has reportedly decreased over the last decades [4,5]. On the other hand, heavy rainfall events have become more common [4,6,7] and are projected to become more frequent in the future [8,9]. According to climate change projection models, rainfall might decrease by 25% in semi-arid regions of sub-Saharan Africa due to future climate change [10].

For millennia, droughts have been common in the arid and semi-arid regions of East Africa due to climate variability caused by the El Niño–Southern Oscillation and other large-scale climatic processes. Such droughts can have devastating impacts on the environment, agriculture and livestock and, therefore, on local economies, contributing to social instability. Low adaptive capacity to droughts makes the region highly vulnerable to climate change [3,6,11,12,13,14,15,16].

Evidence suggests that African ecosystems (e.g., freshwater, forests and grasslands) have already been affected by climate change, and they are projected to be severely impacted in the future. In particular, the availability of water, already a limited resource, will be threatened, limiting economic development of the continent [3].

Health-related vulnerability to climate change depends on environmental, economic and societal contexts, including baseline climate, health system infrastructure, disaster preparedness and general socio-economic resources, as well as on individual factors (education, income, health status) [17]. According to the Intergovernmental Panel on Climate Change (IPCC), health problems that are already in existence will be exacerbated by future global climate change. The burden will be largest on populations, which are already struggling with climate-related diseases, such as malaria and undernutrition [17].

Some manifestations of climate change can impact human health immediately and directly, such as more frequent and severe heat waves that cause heat strokes and other health problems. Other effects are indirect—they are mediated by ecosystems and include, for example, vector-borne diseases due to the proliferation of mosquitoes in a warmer climate and water-borne diseases due to contamination of drinking water after heavy rainfall [17]. Research on such direct and ecosystem-mediated health effects of climate change and weather has increased substantially over the last few years, showing a rich body of evidence from all regions across the globe [17].

However, many effects of climate change on human health are mediated by economic and societal impacts, although these are generally difficult to study because of the complexity of multiple underlying factors. In low-income settings, such as sub-Saharan Africa, adaptive capacity is low, and climate change functions as a multiplier of existing health vulnerabilities related to food insecurity and insufficient access to clean water, education and health care [3]. This is the case for droughts and decreased rainfall which cause harvest failures that threaten the livelihoods of pastoralists and farmers [13,16,18]. In Africa, temperature increases and periods of failing rain have been linked to decreased harvest yields, food shortages and increased food prices and, consequently, to undernutrition, in particular among young children. A possible increase in malnutrition rates, stunted growth and impaired cognitive development has been projected to occur during this century [3,4,19].

Food insecurity can contribute to socio-political instability [20]. The link between climate extremes, political unrest and violence is unclear. On the one hand, evidence indicates that the consequences of droughts, such as soil degradation, water scarcity and overcrowding, can trigger violent conflicts, particularly in politically unstable regions [17]. On the other hand, there is also evidence for decreased conflict levels during drought years in Kenya [14].

Indirect health effects mediated by societal drivers are likely to be profound and prolonged. One example is health hazards related to large-scale migration and forced displacement resulting from extreme weather events, either within the country (internally displaced people, or IDPs) or cross-border [19,21]. Displacement might occur temporarily (e.g., after a flood) or it may be long-term or permanent, in particular when rural livelihoods fail [22,23]. There is a good understanding of the scope and health consequences of displacement in the course of sudden-onset disasters such as floods. Droughts and other slowly progressing natural hazards are, however, difficult to link to migration, human security and health, owing to the mediating roles of the socioeconomic, political and environmental context. So far, there has been only limited empirical research in this field [24,25].

Climate change can trigger displacement, but migration can also be a successful means of adaptation [20]. According to the IPCC, there is some evidence that future climate change will increase displacement rates, in particular among poor populations in low-income countries [23]. Sea level rise, coastal erosion, impaired agricultural yields and other environmental changes are contributing factors, although causal attribution is difficult to establish [20,24,26]. In sub-Saharan Africa, the combined pressures of population growth, land degradation, wars, food crises and droughts have been named as forcing environmental migrants abroad in search of better livelihoods [22]. The World Bank has, for example, projected an increase in climate-driven migration in Ethiopia over the next few decades—up to 1.5 million people by 2050—mostly caused by water scarcity [21].

To some degree, internal and cross-border displacement is caused by climate-related violent conflicts and political instability in this region [22,23].

Sub-Saharan African has a large rural population, with two-thirds of the workforce employed in agriculture, which is thus a key income source [10,19]. Since many regions are facing scarce and unreliable rainfall and are reliant on rain-fed agriculture, the population is highly vulnerable to climate change [10,19,21]. Decreases in crop yields and increases in food prices pose an economic threat and might lead to conflicts and political instability in many sub-Saharan countries [21].

The Greater Horn of Africa, at the boundary of the Sahel in north-eastern Africa, encompasses three countries: Somalia, Kenya and Ethiopia. Somalia and Ethiopia are classified by the OECD (Organisation for Economic Co-operation and Development) as least developed (low income) countries; Kenya recently obtained status as a lower-middle income country [27]. These are, thus, poor countries, with a substantial part of the population living close to or below the poverty line. Despite rapid urbanization, a majority live in rural areas, earning their main income as pastoralists or small-scale farmers [28,29,30].

The region has a long-standing history of political unrest, large socio-economic differences and poor population health. Against this background, climate change has the potential to aggravate existing health challenges, as adaptive capacity and climate resilience is overall low.

Eastern Africa has a predominantly arid or semi-arid climate with two rainy seasons and considerable inter-annual variations in rainfall [3,31]. Droughts have been a common phenomenon for centuries, to which people have adapted by seasonal migration and extensive agriculture. However, increases in droughts and heavy rainfall pose severe limits to adaptation. The droughts of 2011 and 2017/2018 were particularly severe and led to famine, high levels of malnutrition in children under five and mortality in most parts of the three countries [28,32,33].

Under these precarious environmental and socio-political circumstances, migration becomes a necessity for survival for hundreds of thousands of people [34]. This is the background for the present study, which aimed to investigate the health impacts of drought-related migration and health care access in Somalia, Kenya and Ethiopia. Specifically, we assessed knowledge status and knowledge gaps regarding public health implications of large-scale migration in this region, both for the displaced people themselves and for the host communities, including health status and health care needs of displaced populations.

## 2. Materials and Methods

### 2.1. Study Team, Study Design and Data Collection

The ‘ClimRef’ project was conducted during 2018 by a team of three public health scientists from Sweden, supported by eight country partners—researchers and decision makers based in Somalia, Kenya and Ethiopia with expertise in health sciences (public health and medicine) and climatology.

Detailed methods and results of the project are described in three country reports [35,36,37]. The first component of the project consisted of a scoping review of the published literature, while the second component, a knowledge assessment using qualitative methods, was conducted among stakeholders and decision makers in the three countries. This article presents the findings of the second component.

In this study, data collection was undertaken in two phases. The first phase included qualitative in-depth interviews conducted between April and May 2018. The second phase included a stakeholder workshop in October 2018, in which the preliminary findings from the in-depth interviews were discussed.

The interview guide that was used was based on the aforementioned scoping review as well as on the pre-understanding of the team members. It was developed by the Sweden-based researchers and was sent out to the entire team for feedback. The finalized interview guide included questions on the following areas: health status and health care needs of drought-related migrants; health care access for refugees and IDPs; climatic and environmental changes; suggested good practices; and future needs for building resilient health systems.

Country partners identified from their own networks and contacts potential interviewees in each of the three countries. Interviewees were purposively selected based on their expertise in one or more of our core topics of concern: climate, weather, environment, migration and public health. Participants also represented different institutions and agencies in different areas at the national, regional or local level, including governmental agencies, non-governmental organizations and UN agencies. In total, 46 experts were invited to participate in the interviews.

The complete interview guide (Supplementary Materials) was sent to the participants by email prior to the interview, although in the interviews, they were asked only those questions that related specifically to their particular area of expertise. The different issues were explored both at the national level and at the regional level, depending on the expertise of the interviewee.

All interviews were conducted by two of the three Sweden-based researchers. One researcher led the interview while the other took extensive notes of what was said. The country partners organized the interviews and, in some cases, they also participated. Interviews with Somali respondents were conducted via the internet; Kenyans and Ethiopians were (with two exceptions) interviewed face-to-face in Nairobi, respectively Addis Ababa and Mekelle (Tigray Region). Interviews lasted between 30 to 60 min. Verbal informed consent was obtained at the start of each interview and anonymity for all interviewees was guaranteed. Following each interview, the two researchers who conducted the interview had a short discussion about emerging themes and to clarify any uncertainties that may have arisen. In addition, the researcher who asked the questions cross-checked the notes of the note-taker.

### 2.2. Analysis

Notes from the interviews were imported into NVivo software, version 11 (QSR, Melbourne, Australia) and subjected to thematic analysis [38,39]. During the first step of analysis, researchers familiarized themselves with the entire dataset. This was followed by an initial generation of codes and a sort of the material into themes that were largely based on the pre-determined themes of the generic topics covered by the questions. The analysis was separately conducted for each of the three countries, primarily by the second author, but in close discussion with the first and the last author (KL and BS). All themes were therefore discussed and negotiated throughout the analyses.

A draft of each country report containing the preliminary results from this analytical process was disseminated to the interviewees and country partners for that respective report. After this, the results were presented and discussed at a validation workshop held in Nairobi in October 2018. A total of 13 delegates participated (eleven interviewees, two country partners and two Swedish researchers), three of whom were from Somalia, six from Kenya and four from Ethiopia. Extensive notes were taken during the group and plenary discussions, and the critiques and suggestions that emerged were then incorporated into the next phase of analysis. No new themes, however, emerged from this phase.

In the final stage of analyses, findings from the three countries’ datasets and from the validation workshop were analyzed as one dataset. In this analysis, the focus was on constructing cross-cutting themes from all three settings. It was conducted by the first author (KL) in close discussion with the second and the last author (JK and BS).

### 2.3. Ethical Considerations

After a consultation with the country partners, ethical clearance was not sought since no personal information was collected. Participants were interviewed purely in their professional role. The informants were, however, assured that data would be anonymized and that no names would be included in any publication.

## 3. Results

A total of 39 people were interviewed from the three countries (Table 1), including professionals from a range of different institutions: UN agencies, government ministries, non-governmental organizations (NGO) and regional leaders. Thirty of the interviewees were male and nine were female (Table 1).

Three themes emerged from the analysis, which represented the knowledge status and needs of migrants in host communities and camps in Somalia, Kenya and Ethiopia with regard to the public health impact of drought-related migration and access to health care. The themes were (1) health care status and health care needs; (2) access to health care; and (3) needs assessment, preparedness planning and program evaluation.

### 3.1. Theme 1: Health Status and Health Care Needs

For all three countries, there are both highly visible as well as invisible needs faced by people who have been displaced by drought or other hazards. Visible needs include vitally important issues, such as malnutrition and lack of vaccination, which can be measured more easily. Invisible needs, on the other hand, refer to the less easily measured yet still severe conditions. Two of these, raised by interviewees in all three countries, were mental health and gender-based violence (GBV). It also emerged that knowledge about the needs of IDPs was far less extensive than that of refugees.

#### 3.1.1. Somalia

One major health issue that was recognized in Somalia in general and, in particular, among IDPs is the very high maternal, child and neonatal mortality rates. One of the stated causes is the shortage of trained nurses and doctors, especially for migrating people. This shortage occurs in part because many health staff start their own private facilities as for-profit businesses, thereby leaving the public health services understaffed.

Another concern raised by the interviewees was the need to increase vaccination coverage rates. One interviewee reported concerns among some people that vaccination is ‘un-Islamic’, contributing to dangerously low coverage rates in parts of the country.

Mental health is also a great concern that has so far not received much attention. There are innumerable people in Somalia who have been traumatized by war and other forms of violence, including GBV or forced displacement due to war or climate change. In addition, poor sanitation was stressed by several interviewees as a source of serious concern in IDP camps across the country.

#### 3.1.2. Kenya

In Kenya, pastoralists and nomads were identified as being extra vulnerable to the impacts of climate change, and hence they exhibit specific health care needs. For example, Mandera County, as in most pastoral counties in the country, is dominated by pastoralists and is highly susceptible to droughts. It also has the highest rates of malnutrition, under-five mortality and maternal mortality in the country. In addition, livestock can be lost during a drought and this has a serious long-term effect on a community’s economy and, in turn, also has an adverse effect on health. Human–wildlife interactions during a drought can increase the risk of zoonotic disease transmission.

By contrast, floods in the lowlands have caused significant damage to houses and crops, with a corresponding loss of food and other belongings, and this has led to an increased risk of vector-borne diseases, such as Chikungunya, malaria, Rift Valley fever and kala azar (visceral leishmaniasis). At the time of our interviews (late April 2018), around 150,000 people had been displaced by floods. Water-borne diseases such as cholera can also emerge; and snakebites may become a major problem as the snakes try to find alternative places to stay. For example, people in Lamu, a coastal location, suffered an unusual number of snakebites during early 2018 due to the high water. Furthermore, it can be challenging for farmers to plant after a flood, which means that the effects of the deluge can last for months or more, thereby triggering migration. The nutritional status of children can also be seriously affected because of poor harvests and increased food prices after a flood.

Large indirect effects of climate-related migration may be seen in the urban areas where people relocate. In these settings, the migrants may be faced with the challenge of poor housing coupled with overcrowding and dirty sources of energy for cooking, lighting and heating. This can result in high levels of household air pollution, which may, in turn, lead to increases in cases of chronic obstructive pulmonary disorder, allergies and asthma. Similarly, overcrowding and poor living conditions can contribute to the spread of tuberculosis.

#### 3.1.3. Ethiopia

In Ethiopia, several public health issues were highlighted by the interviewees as having either a direct or an indirect relationship with climate change. These could be applicable both to stable populations as well as to migrants. For example, due to climatic and environmental changes, there is a risk of a resurgence of malaria in parts of the country. In addition, the ecological range for the sand fly is also changing, bringing leishmaniasis into new parts of the country. Drought has also affected large areas of Ethiopia over the past years, which has led to widespread malnutrition that particularly affects children under the age of five as well as pregnant and lactating women.

People who have been forced to migrate due to climate change tend to move from rural to urban areas. These locations are often overcrowded, and they are also places where open defecation is common and the water and sanitation systems do not always function. Consequently, residents face increased challenges with hygiene and sanitation and all of their associated health risks. Poor mental health as well as teenage pregnancies are also significant problems in such settings where displaced people settle.

### 3.2. Theme 2: Access to Health Care

This theme reflects the stakeholders’ perceptions regarding access to health care for displaced people in the three countries. Challenges raised by the interviewees in all countries included lack of health care services for mental health and GBV, possible tensions over access to care between migrants and host communities and the additional vulnerability of pastoralists in terms of limited access to health care.

#### 3.2.1. Somalia

There is uneven knowledge about access to health care in Somalia. For example, since NGOs and health care agencies tend to be sited in more accessible urban areas, rural areas and conflict-affected areas remain underserved, and knowledge of the health status and health care needs of people living in rural and conflict areas, including people migrating due to drought, is limited.

Health care services in Somalia are provided by NGOs, by the government and by the private for-profit sector. The health infrastructure was described by one interviewee as ‘extremely bad for host communities, but it’s even worse for IDPs’. Coordination between the different categories of providers is also not always optimal, and we were informed of poor regulation of private sector health care providers. Still, people, including IDPs, tend to perceive the services of the private for-profit sector as better than those provided for free. IDPs living in established camps have full and free access to basic public health services, including vaccinations and maternal and child health care. However, surgical issues and non-emergency examinations and procedures are not free of charge, and these are provided largely by the private sector. Since IDPs are often extremely poor, many do not have access to such services.

One general challenge is the level of education among the health workers who are responsible for much of the primary health care system in the country. As they have a rather low level of education, they are unable to provide a technically desired level of service.

It was noted during the interviews that there are virtually no mental health services or services for GBV, and we were informed that there has been no substantive study of any sort to assess this. Donor agencies are keen to focus primarily on visible, life-saving issues, such as reproductive, maternal, neonatal and child health; sanitation; vaccination; and nutrition interventions, in which outcomes can be easily measured and, consequently, resources can be allocated. Without an empirical foundation, there will be no political imperative to raise mental health as a major issue that needs to be addressed; providing mental health services (pharmaceutical treatments, therapy or counselling) is not prioritized by the donors, since it is not considered to be directly saving lives. As one interviewee expressed, ‘Donors do not see the value for money in this issue… The donors need to see a tangible effect for their money. For example, it’s easier to show an effect of a nutritional program by measuring upper arms’.

Another serious factor was aid dependency since Somalia’s health care is based on an agenda that is largely set by the donors and does not always fully reflect the population’s needs. As one of our interviewees said, ‘The UN [and other agencies] are just putting a Band-Aid on a larger problem’, and this does not sufficiently address the issues at hand. The country is also vulnerable to donor fatigue, such as during the 2017 drought when funds for humanitarian support were insufficient.

#### 3.2.2. Kenya

In Kenya, knowledge of the health status of cross-border refugees and migrants is relatively comprehensive, and this may facilitate access to health care. Upon arrival at formally designated camps, refugees are supposed to undergo a standardized, systematic health screening: people are checked for diseases with epidemic potential, including viral hemorrhagic fevers, as well as for malnutrition. Mothers are asked about their children’s vaccination status. The health authorities are cognizant of the sub-optimal vaccination status of refugees from Somalia and thus of the need for extra effort when handling these refugees. There is extensive polio surveillance due to new cases reported in neighboring Somalia and South Sudan in recent years. Further, to ensure continuation of HIV and tuberculosis (TB) treatment that started in the home country, counsellors hold confidential conversations with refugees about their disease status and treatment of these diseases is provided for free.

One new initiative started by the World Health Organization (WHO) and NGOs is a cross-border coordination mechanism between Kenya, Uganda and South Sudan, with the aim of improving coordination between the health services of neighboring countries regarding disease outbreaks or other events in nearby areas and on migration routes.

There is very limited knowledge about people’s mental health care needs and, consequently, little in the way of the provision of mental health services for refugees and IDPs or for the local population. Training for health workers on mental health was also highlighted as an important knowledge gap.

A significant proportion of the Kenyan population practice a nomadic or semi-nomadic pastoral lifestyle, and they are highly mobile, irrespective of climate-related concerns. Rural areas where pastoralists live exhibit weak health infrastructure, and mobile clinics as an alternative to cope with their pastoral lifestyle are also limited. All these factors compromise the health of pastoralists and thus further dispose them to poor health.

#### 3.2.3. Ethiopia

One general concern raised by some interviewees was the relative lack of connection in terms of planning between the health system and climate change. One interviewee expressed that the authorities have been responding to climate extremes ‘like firefighters’, reactively rather than proactively. This interviewee also stated that the link between health systems resilience and the climate has only very recently been recognized and the thinking is not yet built into the system. Connected to this was a concern raised about the challenges in advocating for family planning services as a means of establishing ‘better family sizes’ so that it would be more feasible for people to feed themselves during periods of food shortage.

The relationship between refugees and host communities can affect access to health services in both positive and negative ways. Eritrean and Ethiopian people are ethnically similar to one another, and this facilitates integration and good relations between Eritrean refugees and the Ethiopian host communities. Nonetheless, tensions have still arisen in Tigray, in the north of the country, when Eritrean refugees have cut trees for fuel. Interviewees expressed the importance of enabling a shared responsibility for the limited natural resources, such as firewood. As one interviewee stated, people in the host communities sometimes complain that, ‘We are planting but the refuges are cutting. We are taking care of the environment but the refugees are not’. In other circumstances, the resentment caused by such practices can also be applied to IDPs, leading to social exclusion and, in turn, a reduction in access to health services by displaced population groups.

Further, since the precise number of IDPs in any given place is rarely available, it can be difficult for authorities to project sufficient drug supplies or to provide suitable sanitation, antenatal care, vaccination programs and mental health services, as well as support for women suffering from GBV. Although refugees are screened upon arrival at a camp in order to assess overall health and nutritional status and to provide measles and polio vaccinations as necessary, there is no such screening provided for IDPs, and so their specific requirements are unknown.

### 3.3. Theme 3: Needs Assessment, Preparedness Planning and Programme Evaluation

The third theme that emerged from our interviews concerned health care needs assessments and program evaluation. Specifically, these were said to be limited both in extent and quality in all three countries. One of the overarching messages expressed by the study participants was that program evaluations should include a focus on patient experiences with regard to access, availability and acceptability, and that evaluations should also incorporate a local perspective into policy and planning. A national level health systems perspective is not sufficient to meet the needs of people in the community, and health services at the local level need to be properly evaluated.

#### 3.3.1. Somalia

A major limitation of the humanitarian work in Somalia is that little of it is based on a clear understanding of what the target communities actually need, and little of the work is comprehensively evaluated. One challenge of continuously reaching out to the population in humanitarian need is the fact that it can be physically dangerous to conduct monitoring and evaluation exercises. For example, the numbers of recipients reported on a specific intervention may be inflated by local leaders in order to continue to receive financial support from a donor, and it may be dangerous for an external evaluator to report this. One interviewee expressed this as follows, ‘If you don’t want to have any friends in Somalia, join M&E [monitoring and evaluation]!’ In addition, when internal evaluations are conducted, many NGOs reportedly focus on communicating success stories rather than challenges in their work. This means that lessons are often not systematically learned, and similar mistakes may be made again in the future. However, some NGOs do highlight challenges, possibly as a means of identifying areas for the donors for future dedication of resources, thereby ensuring a continued flow of money for their own humanitarian scope of work.

In spite of these concerns, we were informed of serious efforts at conducting needs assessments with a user perspective by at least one NGO interviewee to guide their work in the IDP camps. Two methods described were: (i) meeting with community representatives, and (ii) suggestion boxes with suggestions given on feedback forms. However, both of these methods have challenges in that community representatives wish to promote issues that may benefit specific groups (for example, based on kinship patterns), so that the information provided may be biased in that direction. The NGO, therefore, verifies information to the extent possible by speaking with other spokespeople, for example, for youth, women and the disabled. Regarding the suggestion boxes, many people are illiterate and are either unable to fill in the forms, or they get help from people who may then write about their own concerns. Another method of learning about community concerns was a free hotline in an IDP camp that people could call. The topics raised could then be collated and key issues of concern identified.

#### 3.3.2. Kenya

The Climate Change Act 2016 defines the key institutions responsible for coordination and implementation of climate change actions in the country. These are the National Climate Change Council chaired by the President, the Climate Change Directorate (CCD) and the climate change units at national and sub-national level governments. The climate change units coordinate climate change planning, budgeting and mainstreaming at all sectors. These institutions cut across all sectors, including those handling issues of health and migration. The CCD developed the National Climate Change Action Plan 2018–2022, which highlights priority actions in seven strategic objectives. The National Climate Change Action Plan is the main tool used to propel the country to a low carbon climate resilient development pathway.

All climate change policy documents in Kenya underscore the need to mainstream climate change in all sectors. Refugees needs cut across all social economic sectors, which requires that all sectors are climate proofed.

Each county in Kenya has a County Steering Group (CSG), which includes all main actors at the county level. This provides an institutional arena for the discussion of current issues and needs and for the dissemination of any plans that may result from these discussions. The value of this structure was shown in the drought of 2016/17, when fodder was effectively supplied to people for their starving animals, thanks to good partnerships and coordination from the national to the community level.

One critical issue that needs to be addressed when large numbers of migrants are arriving in an area is the needs of the host population. While the migrants may be accorded priority to services because of their situation which is inherently more acute, we were told that the host populations should also receive some sort of benefit, especially if the host community is also equally vulnerable, in order to reduce possible conflict over limited resources.

The main knowledge gaps identified by our interviewees were found to be at the local or community level. Since decisions about the provision of public health services must resonate with the local context, effort should be made to facilitate the collection of relevant local data. This process requires that the county authorities identify and request better and more specific data so that they can optimize service delivery. The interviewees particularly expressed the need for a more in-depth understanding of the migrants’ thoughts and experiences gained, for example, by the use of needs assessments and evaluations focused on issues such as access to and acceptability of the services provided.

#### 3.3.3. Ethiopia

Systematic efforts to evaluate health services for refugees in Ethiopia are made by some partners, such as UNHCR (United Nations High Commissioner for Refugees). These evaluations are primarily conducted from a health system perspective, with a focus on, for example, intervention coverage rates. The community perspectives of a given service (e.g., concerning access, availability and acceptability) are rarely investigated. Evaluations are periodically conducted on refugee services (though rarely on services for IDPs), including on patient satisfaction, the utilization and non-utilization of services and costs incurred. Through this, operational lessons can be learned.

In spite of these mostly ad hoc efforts, there is insufficient overall knowledge about the health care needs of displaced populations in the country. The integration of health care services in the refugee camps with those serving the rest of the local community also appears insufficient and requires further evaluation. A challenge in preparedness planning is that services for the camps are run by UNHCR while services for the local communities are run by the Ministry of Health. This can lead to inequalities in service provision between the two communities with the subsequent potential for conflict.

## 4. Discussion

The Horn of Africa is affected by chronic food insecurity and mass displacement, both of which are triggered, to some degree, by droughts and related environmental hazards [40]. These challenges have the potential to amplify existing issues of political instability, weak state institutions, regional conflicts, widespread poverty and social inequalities. In many instances, it is not possible to disentangle the different triggers of displacement. Currently, food security is threatened by a massive invasion of desert locust swarms, which might be linked to climate change [41]. Positive effects of climate change in Somalia, Kenya and Ethiopia are negligible—long-term climate change adaptation planning is paramount. When food systems fail and political and economic systems are insufficient to provide alternative livelihoods for rural populations, migration becomes increasingly necessary for survival.

Our study aimed at identifying the status of knowledge regarding health and health care needs of populations displaced by droughts and related environmental hazards in the Horn of Africa. While we found context- and country-specific areas of concern, there were also several issues that applied across the region in each of the countries of Somalia, Kenya and Ethiopia.

### 4.1. The Unknown Group: Internally Displaced People

A cross-cutting finding in all countries was that there is less knowledge on IDPs as compared to that for cross-border refugees. Although there are a large number of case studies and reports about the situation in refugee camps such as Dadaab (Kenya), our interviewees noted the lack of information on health status, needs and vulnerabilities among those who have been displaced within their own country. Many IDPs move around or stay for shorter periods of time in informal settlements, so authorities have little knowledge of their whereabouts and their needs for basic health care.

One particularly vulnerable group are nomadic and semi-nomadic pastoralists, whose livelihoods, migration patterns and well-being are threatened by environmental changes that might transform planned seasonal migration into de-facto displacement. Their lack of knowledge about health care options as well as their mobility are barriers to their health care access.

Apart from IDPs, we also lack knowledge about the situation of undocumented refugees and migrants. According to a recent investigation by the Norwegian Refugee Council and the International Human Rights Clinic at Harvard Law School in Kenya, many refugees in Nairobi are undocumented, which severely limits their access to health care and other services [42]. Even a survey among migrants in Nairobi found a number of barriers to health care for non-local residents, despite the Kenyan constitution’s provision of equal rights to health for all people in the country. Migrants have reportedly faced health care fees up to ten times the fees for locals, have not always received necessary medication to which they were entitled, and reported other forms of perceived or actual discrimination by health care staff. Documented refugees had better access to health care than irregular migrants [43].

The health impacts of internal displacement, in particular in informal settlements with poor living conditions, are considerable—both direct (injuries and violence) and indirect (malnutrition and communicable diseases). IDPs are disadvantaged not only due to their high mobility but also because in these settlements, they do not have access to services provided for refugees by international aid organizations unless their governments explicitly ask for this support. This includes the provision of preventive services such as vaccination programs and basic maternal and child health care. However, while a number of case studies are in existence on specific health issues, such as malaria, malnutrition and mental health, we lack a more detailed picture of health status and health care access of IDPs [44].

A positive effort initiated in 2019 in Somalia was the setting of a national policy aimed at durable solutions for the IDPs, starting with the capital Mogadishu where the largest number of IDPs are concentrated [45]. This strategy is supported by international partners by offering land tenures or rental subsidies to IDPs.

### 4.2. The Unknown Trauma: Mental Health and GBV

One major knowledge gap expressed by stakeholders in all countries concerned mental health status and the scope of GBV. The need for services for IDPs and cross-border refugees affected by these traumas is tremendous, while provision of support utterly insufficient. Studies have shown that female IDPs are reportedly at high risk of GBV, which can cause sexually transmitted diseases, unwanted pregnancies, physical injuries and psychological trauma [44]. Children who have been displaced by drought are a particularly vulnerable group; they risk separation from their family, psychological trauma and GBV [46].

The current literature shows that mental health needs of refugees are complex and depend on a range of factors before and after migration. For Somali refugees in Nairobi, the intersection of war trauma and daily stressors posed mental health risks [47]. In Melkadida camp, Ethiopia, more than one third of Somali refugees reportedly showed symptoms of depression. Women, those lacking shelter and refugees who had seen family members killed were at particularly high risk of depression [48]. According to a survey among IDPs in Benadir, Somalia, drought, conflict, displacement and harsh living conditions have strong impacts on psychosocial well-being. Many of these IDPs were traumatized by experiencing armed conflict and multiple displacements. Women reported fear of rape and sexual violence, while men’s mental health was, in particular, negatively affected by loss of employment and livelihoods [49]. A Kenyan study among people displaced by violence conducted after the political crisis in 2007/08 revealed extremely poor mental health, life satisfaction and overall quality of life, and many participants showed signs of depression or expressed suicidal thoughts. Anxiety and distress related to the lack of governmental health support and worries about one’s children were common [50].

Apart from case studies and the statements of our informants, there is little empirical evidence about the prevalence of psychological trauma, mental health problems and health care needs of IDPs and refugees in the Horn of Africa. A situational analysis of mental health in Somalia conducted in 2010 estimated approximately 33% of the population was affected by ill mental health. This was also suggested to be higher than in other low-income and so called ‘war-torn’ countries [51]. Unfortunately, mental health has a very low priority for health care planning, which focuses on communicable diseases. In Kenya, less than 1% of the national health care budget is spent on mental health [50], and there is a lack of trained psychological and psychiatric health care staff in the East African region [52,53]. In particular, health care for severely traumatized refugees requires qualified staff, which is currently insufficient [54]. It also requires a good understanding of culture-dependent distress and perceptions and expressions of trauma and distress [55].

### 4.3. The Unknown Place: Knowledge of Local Conditions for Decision Making

The importance of including the perceptions of the local people who are the beneficiaries of humanitarian work was highlighted in all three countries as an area where significant improvements are needed. The voices of communities are seldom heard in national and regional level discussions about interventions and solutions, and their in-depth knowledge of local conditions is not considered. The ideas and interventions that are planned and implemented are, therefore, not always well-grounded in local conditions, and they do not necessarily meet the core needs of the local people or make good use of the community’s existing capacities and knowledge.

In 2020, an estimated 5.2 million people in Somalia are in need of humanitarian support [56]. The difficulties in understanding their needs is related to the challenge of conducting continuous evaluations, in particular of those population groups who reside in insecure and inaccessible areas of the country. IDPs residing in peri-urban localities are more accessible, though meeting all the health service needs is a challenge.

Humanitarian agencies need to further develop a way of thinking that embraces the possibility of genuine dialogues with the community as an essential prerequisite for any successful intervention. One important group that can strengthen dialogue and tailored health care interventions is the local community health workers (CHWs) who could reinforce primary health care systems. The deployment of such health workers in remote rural communities to promote services and lessons about health and climate change would speed up progress towards Universal Health Coverage. One challenge mentioned by Somalian interviewees is the shortage of trained CHWs across the rural areas of the country where they could make a significant difference through the simple messages and the primary health care services they provide.

Another key area is the lack of evaluations of programs and interventions. A recently published study on the humanitarian health response for women and children in Somalia showed that available quantitative data on intervention coverage is very sparse, making it difficult to detect temporal or spatial patterns [57]. Underlying sociocultural and other contextual factors that affect the humanitarian health response included clan dynamics and female disempowerment. Furthermore, the assessment of the needs of the population, donor priorities and insufficient and inflexible funding influence health interventions. Key barriers to service delivery are chronic commodity and human resource shortages, poor infrastructure and limited access to highly vulnerable populations [57]. Migration itself is a very old practice: it is itself a means for coping with environmental hazards and economic hardship [16,58]. Climate change has brought this to the discussion in a new way, and the limits of migrational adaption have become obvious. There is a need for a better understanding of the existing capacities in the migrating communities before introducing adaptation plans at the national or regional level. This can be particularly applicable for nomadic people who have rich experience in coping with droughts by seasonal migration. Indigenous knowledge (IK) is the insight of local people that enables them to make a living in the specific requirements of a certain environment [59,60,61]. Unfortunately, there has been a loss of IK and its practices among indigenous communities in the last decades [62,63]. This is important to counteract and acknowledge together with modern technologies, given that IK often is innovative and helps to reduce and mitigate risks [64,65]. Pastoralists maintain and carry forward IK and its practices, enabling them to cope with droughts and thus manage both short term and long-term food insecurity [65].

Conducting reliable, community-based needs assessments and evaluations that engage different stakeholders in different steps of the evaluation is key to ensuring that local knowledge is used and that any interventions are feasible, acceptable, and sustainable [66]. Evaluation of services should investigate patient experiences with regard to access, availability and acceptability.

### 4.4. Strengths and Limitations

This is a rather unique study that focused on health issues of ‘climate refugees’—people who have been displaced by droughts or other natural disasters. To explore this, we interviewed a range of stakeholders that could contribute their specific knowledge on climate, weather, environment, migration and/or public health. Participants also represented different institutions, agencies and areas at the national, regional and local levels. Interviews were not recorded, as it was suggested to us prior to the study that participants would not feel comfortable with being recorded (in their professional role). This is a potential limitation in that verbatim transcripts are not available. On the other hand, participants may not have been willing to participate if they knew that they would be recorded, and their perspectives would have been lost. Instead, extensive notes were taken by one of the participating researchers and cross-checked after the interview. The questions were sent to participants beforehand to allow them to reflect on the question areas. This could contribute to richer information in the interviews. It could potentially limit the interviewees, if they felt too restricted by the questions in the interview guide. However, this was not our impression during the interviews, as the interviewees also shared their individual reflections in their answers and allowed for additional probes during the interview. Another limitation of the study was that only nine out of 39 interviewees were female. This may have contributed to a lack of the female perspective. Furthermore, the perspectives of refugees, IDPs and host communities were not captured in the current study.

Measures were taken to increase the trustworthiness of the study [67]. Credibility was secured by inviting participants who represented different experiences. We further strived to increase credibility by presenting our results and receiving feedback from participants both in the preliminary country reports and in the validation workshop. Dependability was considered through the use of regular peer-debriefing sessions with KL, JK and BS, sessions that ranged all the way from the study design to data analysis and interpretation of the results. To increase transferability, we provided extensive descriptions of the context, the selection process of the participants, participant characteristics and the process of data collection and analysis as well as a comprehensive presentation of the findings. Confirmability was supported by having a multidisciplinary research team with both ‘insiders’ (AA, AD, MFA, HG, LLT, MOM, BKM, JM) and ‘outsiders’ (KL, JK, BS) to the study context.

## 5. Conclusions

Our study region of Somalia, Kenya and Ethiopia in the Horn of Africa is constantly facing high rates of undernutrition, morbidity and mortality due to droughts, violent conflicts, and socio-economic instability, and climate change is increasing these risks, further deteriorating the quality of life of millions of vulnerable people. In this regard, public health strategies can enhance resilience against the adverse health effects of climate change. These strategies include surveillance and rapid response to the control of communicable diseases, the protection of vulnerable groups from trauma and violence, and the provision of essential health care, including the deployment of community health workers in remote regions.

Refugees and internally displaced people are particularly vulnerable groups, and climate change will further exacerbate the struggle of displaced populations that experience poor living conditions in overcrowded settlements with inadequate protection from weather extremes and lack of access to health care. In particular, the needs of IDPs are not well known and are poorly met in the three countries. One major knowledge gap that was identified concerned mental health status. The need for services for IDPs and cross-border refugees affected by these traumas is tremendous, while the provision of support is insufficient.

This study collected knowledge status and insights among stakeholders from a range of agencies and organizations. However, to obtain a more complete picture of the health status and health care needs of displaced people, research also needs to include the views of displaced people and their host communities.

A development system that moves beyond emergency response to build long-term resilience and sustainability of the society is urgently necessary [68]. This includes the provision of livelihoods and protection from disasters, giving people the possibility to stay in their home villages and care for their livestock even in times of droughts. As a measure for climate change adaptation, strategies are needed that integrate land and water management and disaster risk reduction for resilient development [3]. In many regions, droughts will become more severe, contributing substantially to migration. In countries like Ethiopia that already have high rates of internal mobility, climate, as a driver of migration, must be addressed in adaptation planning [21]. Most sectors in the three countries have not yet integrated climate change into their health care planning. Cross-sectoral collaboration in working with environmental migrants and other IDPs is limited, and there is a need for better knowledge and approaches to cross-border communication between agencies who deal with migrants and displaced populations with the promotion of strategic policies for the implementation of long-term durable solutions for IDPs.

Given the long-term perspective of climate change and its dramatic impacts throughout this century, and given the multi-factorial drivers of migration and displacement, there is an urgent need for political solutions and robust governance for climate change resilience in East Africa. Further research should be conducted in collaboration between academia and stakeholders. This will provide the necessary knowledge at different geographical and organizational levels, also including the community perspective. The knowledge and data gaps identified in this study pose a barrier for adaptation planning at the regional, national and local level. Much can be learned, however, from indigenous populations who have over centuries adapted to substantial climate variability. Sustainable development needs to consider the experiences, knowledge and perspectives of all population groups in order to create peaceful, resilient societies, and to provide livelihoods and good health for all in the East African drylands.

## Figures and Tables

**Table 1:** Number of respondents interviewed from the three participating countries.

Country	Male: Female	Total
Somalia	7:6	13
Kenya	10:2	12
Ethiopia	13:1	14
Total	30:9	39

## Supplementary Materials

The following are available online at https://www.mdpi.com/1660-4601/17/16/5917/s1. Interview guide.
