# Supplementary material for: Health Status and Health Care Needs of Drought-Related Migrants in the Horn of Africa—A Qualitative Investigation
Source: Int J Environ Res Public Health. 2020 Aug 14;17(16):5917. doi: 10.3390/ijerph17165917 (PMC7459765; doi:10.3390/ijerph17165917)
Supplement: Supplementary file 1 [file ijerph-17-05917-s001.pdf]

Interview guide, sent out to stakeholders prior to the interview

ClimRef Project: Resilient public health in the context of large-scale, drought-related migration in East Africa: Knowledge status and knowledge needs
Interview guide

This project aims to identify the knowledge status and knowledge needs regarding current and projected climatic and environmental changes in East Africa (more specifically Kenya, Somalia and Ethiopia), their impacts on migration, and, subsequently, on public health. We are focusing on climate and drought-related migration, both internally and cross-border. However, if you would also like to share your knowledge and experience about migration more broadly during your interview, you are welcome to discuss the public health needs of refugees in general.

This interview guide is divided into five sections (A-E). Depending on your professional background and/or current position, more emphasis may be put on some sections and questions than others.

A. Background and context

1. Please tell us about your organisation, and the public health services it provides for climate and drought-related refugees.
2. What is the legal framework (i.e. laws, policies, charters and/or guidelines) for providing refugee health services in your country?
3. Do you think that the available information regarding the number and geographical distribution of refugees in your country is sufficient for ensuring that they can all be reached with good public health services? What and where are the knowledge gaps in this regard? To what extent are you able to make decisions despite those knowledge gaps?

B. Health status and health care needs of drought-related migrants

4. What is known about how climate and environmental changes and extreme weather events such as floods and drought affect public health in your region, in particular for refugees? Is there any information that you would want more of but which, as far as you are aware, is not available?
5. Consider the public health issues listed below in relation to the climate and/or drought-related refugees that you meet within your organization (i) How would you describe the current status for refugees of those issues on the list that are of relevance for your work. (ii) What additional information or knowledge might your organisation need in order to provide a better service for these refugees?
    a. Vaccination and maternal health;
    b. Nutrition and household food security;
    c. Sanitation;
    d. Sexual and reproductive health;
    e. Provision of ART and adherence support;
    f. Mental health (i.e. depression, PTSD, substance abuse etc.), including for children and their caregivers;
    g. Gender-based/intimate partner violence;

h. Outbreak surveillance;
i. Any other public health issues that you consider to be relevant.

C. Health care access for migrants in host communities and refugee camps

6. What is known about the impact of drought-related migration on public health services for the host communities in your country? What are the positive aspects, if any, and what are the negative aspects, if any?
7. To what extent and in what way/s are there differences in public health needs for public health services in your country with respect to:
   a. Urban versus rural refugees?
   b. Formal refugee settings/camps versus situations where refugees are living within the host population or in other informal settings?
   c. New versus older, better established camps (e.g. Dadaab or Kakuma in Kenya)?
   d. Refugees who continue to be mobile – either within your country, or through cross-border travel – versus those who remain in one place?
8. Taking into account the following four criteria concerning access, what does your organisation know about refugees' perceptions of the public health services that you provide?
   a. Availability
   b. Geographical accessibility
   c. Financial accessibility
   d. Acceptability

   Would you need more information in order to develop a fuller understanding of refugee perceptions of your services? If yes, what type of information would this be and how would this information best be collected?

D. Climatic and environmental changes

9. Can you tell us what sort of information you and your organization have about the occurrence and effects of climate and environmental changes and extreme weather events such as floods and drought in your country? Is there anything that you think you need to know more about in this regard?
10. To what extent and how is climate-related knowledge communicated between the national climate or meteorological services and public health agencies?
11. What is known about climate and environmental changes and extreme weather events such as floods and drought as potential drivers of migration to or in your region?

E. Good practices and future needs for building resilient health systems

12. To what extent does your organisation have preparedness plans for potential future refugee scenarios (specifically including ones related to climate change)? What information would you need in order to identify and understand potential future scenarios, and thereby to develop more robust preparedness plans?
13. Please describe any good practices or innovations that you know of for addressing the public health needs of drought-related refugees, either from your organisation or elsewhere, and that you consider to be of potential value for other countries and service providers in the East African region.
